# Relationships between Morphological Changes of Lower Limbs and Gender During Medial Compartment Knee Osteoarthritis

**DOI:** 10.1111/os.12529

**Published:** 2019-10-29

**Authors:** Yang Lu, Zhan‐le Zheng, Ji Lv, Rui‐zheng Hao, Yi‐ping Yang, Ying‐ze Zhang

**Affiliations:** ^1^ Department of Orthopaedic Surgery The Third Hospital of Hebei Medical University Shijiazhuang China; ^2^ Department of Emergency Surgery The First Hospital of Qinhuangdao Affiliated to Hebei Medical University Qinhuangdao China; ^3^ Department of Orthopaedics The Second Hospital of Tangshan Tangshan China

**Keywords:** Knee osteoarthritis, Minimally invasive surgery, Morphology, Radiology, Total knee arthroplasty

## Abstract

**Objectives:**

To evaluate the dynamic changes of key morphology indicators of the lower extremities in the coronal plane with progressing medial compartment knee osteoarthritis (KOA) with an emphasis on gender‐dependent regional differences.

**Methods:**

The radiographs of patients with non‐traumatic knee pain and varying degrees of genu varus were reviewed. Radiographs were studied in 1538 lower limbs of 883 consecutive patients who visited our hospital from January to July 2017; all patients had long‐standing anteroposterior image‐splicing radiographs taken of their lower limbs. Morphological indicators of bones and joints that can change the alignment of lower limbs or reflect cartilage wear and soft‐tissue relaxation were selected and measured with the help of picture archiving and communication systems. After comparing the data of different genders, the data of males and females was separated into three age groups, <40 years, 40–60 years, >60 years respectively, and then compared among age groups using the Kruskal‐Wallis and Mann–Whitney *U* tests. Scatterplots of age and all the measurements were drawn to determine the strength of the relations. The Pearson correlation test was performed to reveal correlations of measurements and age.

**Results:**

Femoral bowing angle (FBA) and joint line convergence angle (JLCA) have obvious differences between different genders (*P* = 0.001, 0.000, respectively). This suggests that females have greater femoral curvature and joint space angle than males. Significant differences were found in hip‐knee‐ankle angle (HKA), FBA, distal femoral valgus resection angle (DFVRA), medial proximal tibial angle (MPTA), JLCA, and minimum joint space width (min‐JSW) by age groups in females (*P* = 0.000, 0.000, 0.000, 0.000, 0.003, 0.002, respectively). The difference of mechanical medial distal femoral angle (mMDFA) was significant with *P* values less than 0.05 deemed significant (*P* = 0.030). Significant correlations were found between age and all measurements (*r* = −0.166, 0.253, 0.270, −0.147, 0.089, −0.105, −0.076, respectively, *P* < 0.01). Whereas, the difference in min‐JSW by age group was the only significant one in males (*P* = 0.001), and no significant correlation was found between age and measurements (*r* = −0.107, 0.041, 0.134, −0.067, 0.079, −0.134, −0.098, respectively, *P* > 0.01).

**Conclusions:**

As KOA progressed, both dynamic deformation of lower extremities and degeneration of articular cartilage could be found in females, while no obvious dynamic deformations were found in males. Dynamic deformation of lower extremities was the important feature and the major causative factor of KOA in females.

## Introduction

Osteoarthritis (OA) is one of the most important public health problems in the world. Ten per cent of Americans older than 60 have experienced symptomatic OA^1^, ^2^. The knee joint is one of the most easily involved joints, is one of the main pathogenic factors of lower limb dysfunction in the elderly^3^, and has imposed enormous economic and social burdens on the global community^4^. The incidence of knee osteoarthritis (KOA) is increasing year by year with the aggravation of an aging population and an increase in the global obesity rate^5^.

KOA is considered to be caused by a series of structural and functional disorders in the joint^6^ but there is no particularly effective treatment to improve such disorders. The balance between damage and repair of joint structure is broken, which is the cause of KOA. The main clinical symptoms include pain and stiffness of the knee joint, which leads to lower limb motor dysfunction. The imaging features of KOA include joint space narrowing, osteophyte formation, sclerosis of subchondral bone, and cyst of subchondral bone. KOA can be divided into medial tibiofemoral compartment type, lateral tibiofemoral compartment type, patellofemoral compartment type, and total joint type according to the location of onset, of which medial compartment KOA accounts for the majority of cases^7^, ^8^. Medial compartment KOA refers to the medial compartment of the knee joint as the main affected site, which manifests as narrowing of the medial joint space, erosion of medial articular cartilage, varus deformity of knee joint, and “O” type leg changes^9^.

Radiographic film is the main method for diagnosis and evaluation of medial compartment KOA^10^. Simultaneously, it is the simplest, cheapest, and most widely used tool for the diagnosis and judgment of KOA. Radiological findings included osteophyte formation, narrowing of medial articular space, sclerosis and cystic degeneration of subchondral bone, and malalignment of lower limbs.

Lower limb alignment is an important indicator of load transmission and plays a valuable role in the progress of knee osteoarthritis^11^, ^12^. For primary KOA, varus deformity increases the risk of the occurrence and progression of medial compartment KOA, valgus deformity increases the risk of lateral compartment osteoarthritis, and malalignment of lower limbs leads to the dysfunction of lower limbs^13^. The most classic method for measuring the alignment of lower limbs is to measure the hip‐knee‐ankle angle (HKA), which is measured by full‐length radiographs of the lower limbs covering the entire hip, knee, and ankle joints in the weight‐bearing position. HKA was the medial angle between the mechanical axis of the tibia and the mechanical axis of the femur^14^, ^15^. The mechanical axis of the femur is the connection between the midpoint of the femoral head and the midpoint of the knee joint, and the mechanical angle of the tibia is the connection between the midpoint of the knee joint and the midpoint of the ankle joint. Under physiological conditions, the midpoint of each of the three joints is in a single straight line, and the HKA is 180°.

Sharma *et al*.^12^ suggested that malalignment of the lower limbs, such as varus deformity, would lead to a gradual increase in the load of the medial compartment of the knee joint, which would greatly escalate the risk of the progression of knee osteoarthritis. The theory of “non‐uniform settlement”^16^ suggested that osteoporosis around the knee joint and the supporting effect of fibula lead to non‐uniform settlement of the knee joint, the medial tibial plateau collapsed under long‐term compressive stresses, which lead to alignment varus, more overloads on the medial compartment, degeneration of articular cartilage, and final knee varus and osteoarthritis. It showed that deformation was one of the pathogenic factors of KOA. Based on this theory, the proximal tibial osteotomy is used to remove the fibula support which is the important factor of KOA in the treatment of patients with medial compartment KOA and could effectively alleviate the knee joint pain. Other studies have documented the deformities were not limited to the tibia; femoral mechanical axis varus affected the knee varus to almost the same degree as the tibial deformity^17^. However, the morphology of the proximal knee is seldom noticed. Meanwhile, we found the KOA was identified with greater prevalence in females than in males, as previously proved^18^, ^19^, ^20^, ^21^, ^22^. Moreover, the extent of morphological change of female's lower extremities was more visible. However, there have been few reports on the bone dynamic morphological changes of the entire lower limbs of KOA, nor the gender differences of these morphological changes.

The purpose of this study was: (i) to study the indicators of tibial and femoral morphological changes and their law of dynamic changes; (ii) to discuss the indicators of articular cartilage wear and soft tissue relaxation around the joints and study their changing rules; and (iii) to research the sex difference of the dynamic changes of morphology of lower limbs.

## Materials and Methods

### *General Materials*

All radiographs were taken by two trained radiology technicians with the same machine (General Electric Company, Fairfield, USA). We got these images from picture archiving and communication systems (PACS) (Beijing Tianjianyuanda Technology Co., Ltd., Beijing, China). These indicators were measured with the help of PACS and Digimizer image processing and graphical analysis software (MedCalc Software bvba, Ostend, Belgium, version 4.2.6.0). Each indicator was measured three times by the same observer to find the average value.

Finally, 1538 lower limbs of 883 radiographs were included in the study, including 1187 lower limbs of 684 females and 351 lower limbs of 199 males. The average age was 60.86  ±8.62 years (from 17 to 87 years).

### *Radiographic Assessment*

Due to the confusion of the nomenclature of the angles at present, to facilitate the description, the author mainly refers to Paley's method for naming the alignments and angles of lower limbs^24^, and the rest are called after their current, commonly used names. In view of the elaboration of relevant theories, this paper emphasizes the concept of mechanical medial distal femoral angle (mMDFA) instead of mechanical lateral distal femoral angle (mLDFA), which was its supplement angle.

For ease of description, the following points were defined: Fs was the midpoint of the junction of the lower edge of the lesser trochanter and the femoral shaft; Fd was the midpoint of the femoral shaft 10 cm proximal to the knee joint; the bone between Fs and Fd was defined as the femoral shaft; the femoral shaft was divided into three parts, and the midpoint of the middle‐distal 1/3 junction was Fm; the distal femoral anatomical axis was the connection between Fm and the midpoint of the knee joint; and Fc was the midpoint of the femoral shaft at the middle of Fs and Fd.

#### *Inclusion and Exclusion Criteria*

The study was approved by the ethics committee of our institution and involved no privacy issues. The subjects were patients who visited the orthopaedic clinic in our hospital from January to July 2017 and had long‐standing AP image‐splicing radiographs taken of their lower limbs. The inclusion criteria were X‐ray in weight‐bearing position, with both toes pointing forward and lower extremities without internal and external rotation which was defined as the slight overlap of proximal tibiofibular joint account for one third of the fibulae capitulum^23^. The radiographs of the entire lower limbs should include the complete hip, knee, and ankle joint. Hip‐knee‐ankle angle (HKA) < 180° or HKA = 180°. The exclusion criteria are: (i) Morphological factors, such as HKA > 180°, congenital lower limb deformitie; (ii) Related bone diseases, like femoral head necrosis, fractures or surgery of the lower limb, rheumatoid arthritis, ankylosing spondylitis, acute gout flaring, metabolic bone diseases and so on. (iii) Wrong posture, lower extremities internal and external rotation, knees in significant inflexion, without weight bearing, and so on; (iv) Poor shooting quality, hip, knee or ankle joints were obscure.

### *Mechanical Medial Distal Femoral Angle (mMDFA)*

**mMDFA** was the medial angle between the femoral mechanical axis and the tangent of distal femur. It was used to determine whether the mechanical axis of the femur is in a neutral position. Normal value of the mMDFA = 92° (from 90° to 95°) according to the mLDFA. The mMDFA and the mLDFA are complementary to each other (they add up to 180°).

### *Joint Line Convergence Angle (JLCA)*

**JLCA** was the angle between the tangent of the distal femur and the connecting line of the medial and lateral edges of the tibial plateau. It was the indicator of cartilage wear and ligament relaxation (medial convergence, +; lateral convergence, −).

### *Medial Proximal Tibial Angle (MPTA)*

**MPTA** was the medial angle between the tibial mechanical axis and the connecting line of the medial and lateral edges of the tibial plateau. It was used to determine whether the tibia is in a mechanically neutral position. Normal value of the MPTA = 87° (from 85° to 90°).

### *HKA*

**HKA** was the medial angle between the mechanical axis of the tibia and the mechanical axis of the femur^14^, ^15^. It could be used to determine whether there is varus or valgus in the knee joint; in other words, it was the indicator of mechanical abnormality (knee varus, +; knee valgus, −).

### *Distal Femoral Valgus Resection Angle (DFVRA)*

**DFVRA** was the angle between and the distal femoral anatomical axis and femoral mechanical axis. For most people, a 5° to 6° of valgus was considered to be normal. Kharwadkar *et al*. and Mcgrory *et al*. concluded that a fixed 5° to 6° of valgus cut of the distal femur for routine, uncomplicated, primary total knee arthroplasty (TKA) is safe^25^, ^26^.

### *Femoral Bowing Angle (FBA)*

**FBA** was the angle between FsFc and FcFd^27^ (without bowing, 0°; varus bowing, +; valgus bowing, −).

### *Minimum Joint Space Width (min‐JSW)*

**Min‐JSW** was the minimum distance of knee joints. It was the indicator of cartilage wear. Min‐JSW <3 mm means narrowing of articular space.

### *Categorization of Indicators*

**Figure 1:**
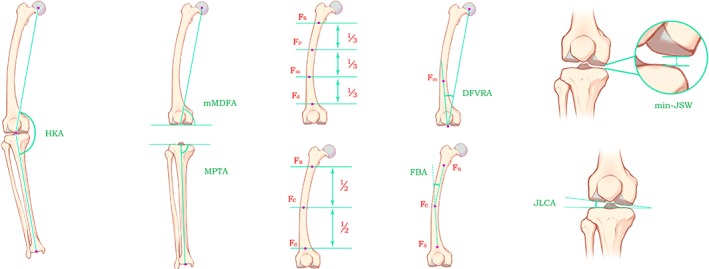
Radiographic measurements: **HKA:** hip‐knee‐ankle angle; **Fs:** the middle point of the junction of the lower edge of the lesser trochanter and the femoral shaft; **Fd:** the midpoint of femoral shaft at the proximal 10 cm of the knee joint; **Fm:** the midpoint of the middle‐distal 1/3 junction of femoral shaft; **Fc:** the midpoint of femoral shaft at the middle of **Fs** and **Fd**; **mMDFA:** mechanical medial distal femoral angle; **MPTA:** medial proximal tibial angle; **FBA:** femoral bowing angle; **JLCA:** joint line convergence angle; **DFVRA:** distal femoral valgus resection angle; **min‐JSW:** minimum joint space width.

Among radiologic parameters, FBA, mMDFA, and DFVRA were the morphological indicators of the femur, MPTA was the morphological indicator of the tibia, and min‐JSW and JLCA were the indicators of cartilage and soft tissue^28^. All angles were showed in Figure 1.

### *Statistical Analysis*

All data was analyzed using SPSS 19.0 version software for Windows (IBM, Armonk, NY, USA). A consistency test was used to evaluate the repeatability of the measurement method. Q‐Q plots and histograms were drawn to check how the data resembles the normal curve. The Shapiro–Wilk test (*W* test) and Kolmogorov–Smirnov test (*D* test) were used to test the normality. After grouping the data, the Kruskal‐Wallis and Mann–Whitney *U* tests were used to assess the differences between the groups. Pearson correlation analysis was used to analyze the correlation of data. Probability values less than 0.01 (two‐tailed) were considered indicative of statistical significance.

## Results

### *Baseline Characteristics*

Finally, 1538 lower limbs of 883 radiographs were included in the study, including 1187 lower limbs of 684 females (77.18%) and 351 lower limbs of 199 males (22.82%). Thirty randomly selected lower limbs were measured again by two researchers with an interval of 1 month to test the reliabilities. Intraclass correlation coefficient (ICC) of main effect indices (we used FBA) which were measured by two researchers and measured twice by the same researcher were calculated. The results showed that all the ICCs were high (>0.9, *P* < 0.01) (Table 1). That is, the measurements of intra‐ and interobserver were reliable and repeatable.

**Table 1:** Consistency check of measurements

Researchers	Measurement 1	Measurement 2	*ICC*	*P*
Researcher A	2.03 ± 2.17	2.03 ± 2.08	0.977	0.000
Researcher B	2.07 ± 2.23	2.10 ± 1.97	0.957	0.000
*ICC*	0.976	0.970		
*P*	0.000	0.000		

The average age was 60.86 ± 8.62 years (from 17 to 87 years). It can be seen that, according to *P* = 0.01 level, FBA and JLCA differ between males and females ( 0.58° and 0.67°, respectively; *P* < 0.01). This suggests that females have greater femoral curvature and joint space angle than males. Angles of different genders are shown in Table 2.

**Table 2:** Descriptive statistics of measured angles

Sex	Age(years)	HKA(°)	Indicators of femur			Indicator of tibia	Indicators of Cartilage and soft tissue	
			FBA(°)	mMDFA(°)	DFVRA(°)	MPTA(°)	JLCA(°)	min‐JSW(mm)
Male	60.49 ± 10.18	173.42 ± 4.54	1.82 ± 2.26	91.13 ± 2.59	5.91 ± 2.04	85.03 ± 2.89	2.66 ± 2.35	2.58 ± 1.68
	(17–87)	(155–180)	(−5–9)	(82–98)	(0–12)	(74–94)	(−4–9)	(0–7.53)
Female	60.97 ± 8.11	172.71 ± 4.91	2.40 ± 2.63	91.30 ± 2.79	6.02 ± 2.12	84.80 ± 3.34	3.33 ± 2.59	2.33 ± 1.48
	(25–85)	(151–180)	(−8–14)	(80–100)	(−2–13)	(70–95)	(−7–12)	(0–7.79)
*P*	0.718	0.020	0.001	0.267	0.321	0.375	0.000	0.016

### *Bisexual Age‐Based Analysis*

**Figure 2:**
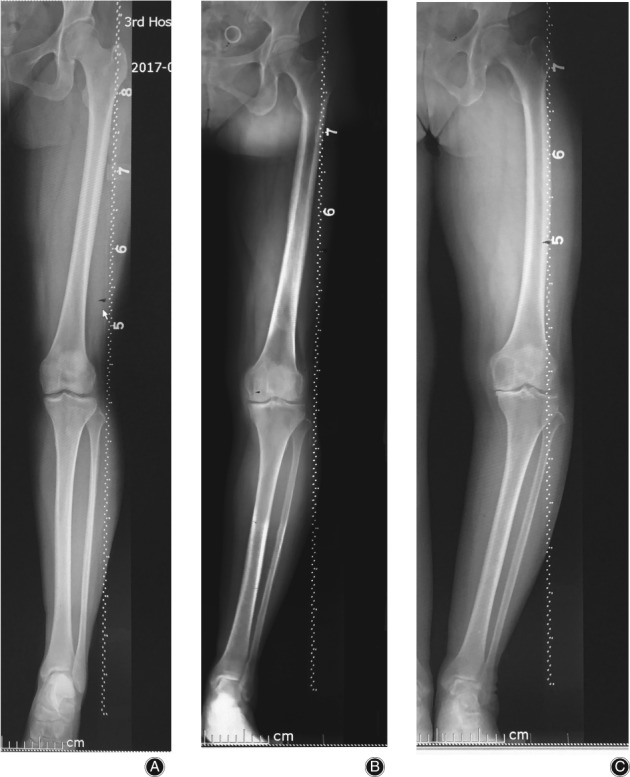
All measurements of lower limbs changed with age in females: (A) Female,37 years old, HKA = 180°, FBA = 0°, mMDFA = 93°, DFVRA = 5°, MPTA = 87°, JLCA = 0°, min‐JSW = 3.50 mm. (B) Female, 58 years old, HKA = 171°, FBA = 1°, mMDFA = 90°, DFVRA = 6°, MPTA = 84°, JLCA = 3°, min‐JSW = 2.82 mm. (C) Female, 66 years old, HKA = 162°,FBA = 8°, mMDFA = 84°, DFVRA = 10°,MPTA = 80°, JLCA = 8°, min‐JSW = 0.7 mm.

**Figure 3:**
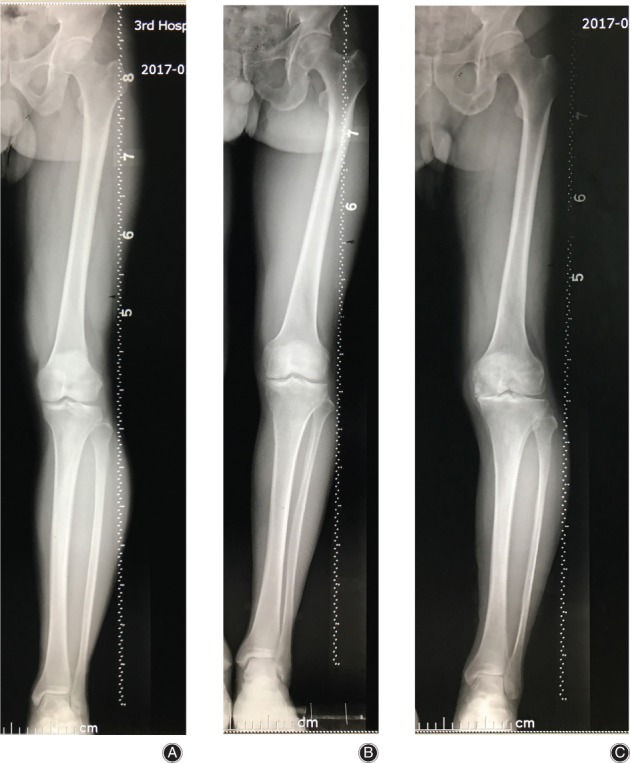
The min‐JSW decreased obviously, but bone morphology changed non‐obviously in males with age: (A) Male, 38 years old, HKA = 180°, FBA = ‐1°, mMDFA = 89°, DFVRA = 3°, MPTA = 92°, JLCA = 2°, min‐JSW = 4.36 mm. (B) Male, 60 years old, HKA = 177°, FBA = 0°, mMDFA = 91°, DFVRA = 5°, MPTA = 88°, JLCA = 3°, min‐JSW = 3.32 mm. (C) Male,72 years old, HKA = 176°, FBA = 0°, mMDFA = 92°, DFVRA = 5°, MPTA = 83°, JLCA = 4°, min‐JSW = 0.00 mm.

Data of males and females was separated into three groups depending on ages of <40 years, 40–60 years, and >60 years respectively. Mean angles within the groups showed some differences. As the data were not normally distributed, a non‐parametric Kruskal‐Wallis test was used to check the difference among groups, and differences were found significant for all variables except mMDFA in females (*P* < 0.01). The difference of mMDFA was significant with *P* values less than 0.05 deemed significant (*P* = 0.03). The >60 group have the smallest HKA, mMDFA, MPTA, and min‐JSW, and the biggest FBA, DFVRA, and JLCA (Table 3). The Mann–Whitney U‐test was carried out between groups in pairs. There were significant differences in FBA, DFVRA, and JLCA between the <40 years group and the 40–60 years group (the change was 1.87°, 2.24° and 2.54°, respectively; *P* < 0.01). Significant differences were also found in HKA, FBA, DFVRA, and MPTA between 40–60 years and > 60 years groups (the changes was −1.35°, 1.33°, 1.03° and − 0.94° respectively; *P* < 0.01) (Table 3) (Fig. 2 shows typical bone morphological changes of lower limbs in female patients at different ages). But in males, differences among age groups were significant only in min‐JSW. Significant differences were found in min‐JSW between 40–60 years and > 60 years groups (the change was −0.49; *P* < 0.01) (Table 4). Fig. 3 shows a diminishing min‐JSW but non‐obvious bone morphological changes in male patients at different ages.

**Table 3:** Mean values of groups (Female)

Groups	HKA(°)	Indicators of femur			Indicator of tibia	Indicators of Cartilage and soft tissue	
		FBA(°)	mMDFA(°)	DFVRA(°)	MPTA(°)	JLCA(°)	min‐JSW(mm)
A	175.25 ± 4.61	−0.17 ± 1.40	92.25 ± 1.35	3.25 ± 1.76	84.92 ± 5.52	0.75 ± 2.22	3.52 ± 1.24
B	173.42 ± 4.53	1.70 ± 2.46	91.50 ± 2.81	5.49 ± 1.94	85.31 ± 3.09	3.29 ± 2.42	2.42 ± 1.39
C	172.07 ± 5.13	3.03 ± 2.61	91.13 ± 2.79	6.52 ± 2.13	84.37 ± 3.44	3.42 ± 2.70	2.24 ± 1.55
*P*_0_	0.000	0.000	0.030	0.000	0.000	0.003	0.002
*P*1	0.090	0.004	0.287	0.000	0.316	0.001	0.013
*P*2	0.000	0.000	0.023	0.000	0.000	0.492	0.019
*P*3	0.017	0.000	0.115	0.000	0.097	0.001	0.006

Group A: age < 40 years; Group B: age from 40 to 60 years; Group C: age > 60 years. Data are expressed as mean ± standard deviation. *P*_0_: P values of Kruskal‐Wallis test by comparing the three groups; *P*1: *P* values of Mann–Whitney *U*‐test by comparing group A and B; *P*2: *P* values of Mann–Whitney *U*‐test by comparing group B and C; *P*3: *P* values of Mann–Whitney *U*‐test by comparing group A and C; *P* < 0.01 was defined as indicating a statistically significant difference.

**Table 4:** Mean values of groups (Male)

Groups	HKA(°)	Indicators of femur			Indicator of tibia	Indicators of Cartilage and soft tissue	
		FBA(°)	mMDFA(°)	DFVRA(°)	MPTA(°)	JLCA(°)	min‐JSW(mm)
A	176.5 ± 3.93	0.25 ± 1.91	92.75 ± 2.55	3.75 ± 2.66	86.38 ± 4.57	2.25 ± 1.28	4.01 ± 1.47
B	174.04 ± 4.07	1.92 ± 2.20	91.30 ± 2.42	5.75 ± 1.97	85.32 ± 2.84	2.36 ± 2.22	2.81 ± 1.68
C	172.75 ± 4.82	1.81 ± 2.31	90.91 ± 2.70	6.15 ± 2.01	84.73 ± 2.83	2.92 ± 2.48	2.32 ± 1.64
*P*_0_	0.013	0.104	0.084	0.019	0.065	0.145	0.001
*P*1	0.129	0.034	0.137	0.028	0.636	0.806	0.043
*P*2	0.022	0.660	0.144	0.131	0.024	0.057	0.006
*P*3	0.037	0.046	0.069	0.013	0.386	0.462	0.006

Group A: age < 40 years; Group B: age from 40 to 60 years; Group C: age > 60 years. Data are expressed as mean ± standard deviation. *P*_0_: *P* values of Kruskal‐Wallis test by comparing the three groups; *P*1: *P* values of Mann–Whitney *U*‐test by comparing group A and B; *P*2: *P* values of Mann–Whitney *U*‐test by comparing group B and C; *P*3: *P* values of Mann–Whitney *U*‐test by comparing group A and C; *P* < 0.01 was defined as indicating a statistically significant difference.

### *Correlations Between Variables*

**Figure 4:**
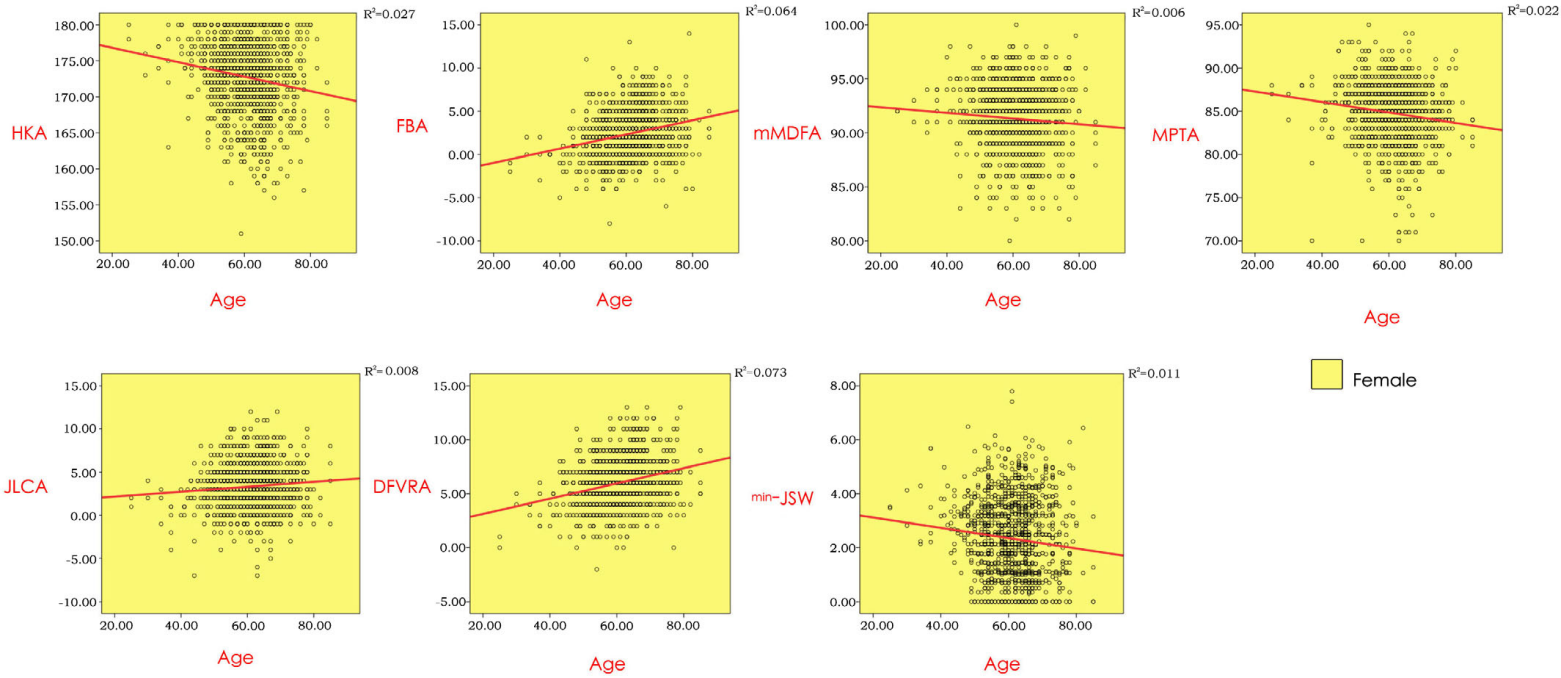
Relations between age and variables (Female). **HKA:** hip‐knee‐ankle angle; **FBA:** femoral bowing angle; **mMDFA:** mechanical medial distal femoral angle; **MPTA:** medial proximal tibial angle; **JLCA:** joint line convergence angle; **DFVRA:** distal femoral valgus resection angle; **min‐JSW:** minimum joint space width.

For females, we pictured some relations between variables by drawing scatterplots to determine the strength of the relations and carrying out correlation tests (Fig. 4). Pearson correlation test was performed to all the variables for they were continuous variables of non‐normal distribution with a large sample size. Table 5 shows there were significant correlations between age and all the other values in females (*P* < 0.01). Correlation analysis was also performed for males, but the results showed no significant correlation (*P* > 0.01) (Table 5). However, significant correlation could be found between age and HKA, DFVRA, and min‐JSW, with *P* values less than 0.05 deemed significant (−0.107, 0.134, −0.134, *P* < 0.05).

**Table 5:** Pearson correlation test between age and measurements

Indexes	HKA(°)	Indicators of femur			Indicator of tibia MPTA(°)	Indicators of Cartilage and soft tissue	
		FBA(°)	mMDFA(°)	DFVRA(°)		JLCA(°)	min‐JSW(mm)
Age	−0.107	0.041	−0.098	0.134	−0.067	0.079	−0.134
(Male)	0.045	0.445	0.068	0.012	0.212	0.137	0.012
Age	−0.166	0.253	−0.076	0.270	−0.147	0.089	−0.105
(Female)	0.000	0.000	0.009	0.000	0.000	0.002	0.000

## Discussion

### *Implications of Indicators*

#### *Morphological Indicator of Tibia*

Knee varus is the morphological manifestation of medial compartment KOA^7^, ^8^. The phenomenon of “non‐uniform settlement” suggested that knee varus started at medial tibial plateau collapsed as long‐term pressure overload led to alignment varus, more overloads on the medial compartment, and degeneration of articular cartilage^16^. Age was an important risk factor for KOA as it increased the length of weightbearing over time^29^. In this study, MPTA was included as the key indicator of tibial morphological changes, for it represents the change of tibial alignment and is the angle needed to be corrected in high tibial osteotomy (HTO) or total knee arthroplasty (TKA).

#### *Morphological Indicators of Femur*

The femur and the tibia participate in the working of the knee joint. After the static loading passes along the femur and travels across the knee center, the conduction was then sent through the tibia shaft to the ankle center. The morphological changes of the tibia should be relative to the ideal tibial plateau (MPTA =90°), and the degree of the medial plateau collapse could well reflect the location and size of the stress. For the same reason, if the femur is deformed, the change of femoral mechanical axis should be compared with the ideal one (mMDFA = 90°). Femoral deformation in medial compartment KOA was proposed for the first time by Cooke *et al*.^30^ Issin Ahmet *et al*.^17^ indicated that the effect of femoral deformation on knee varus was as important as that of the tibia. In our research, we highlight the concept of mMDFA to underscore its unique implications, as the medial distal femur which bore more force was the probable location of the deformity. Given this, we included mMDFA for it represents the change of femoral alignment and is the angle needed to be corrected in femoral osteotomy or TKA.

The existence of femoral physiological anterolateral arch in coronal radiographs embodied as femoral bowing affects the DFVRA^31^. A fixed 5° to 6° of valgus cut angle of the distal femur for TKA of an excessive bowing femur could lead to unsatisfied alignment in the clinic. When compared with the Western population, Asians had bigger DFVRA and greater variation range^32^, ^33^, so individualized measurement of DFVRA should be performed to get a correct distal femoral cutting when performing TKA. Given this, FBA and DFVRA were selected in our research for their important role in TKA or knee‐salvage treatment.

#### *Morphological Indicators of Cartilage and Soft Tissue*

JLCA and min‐JSW were measured for they were evaluation indicators of the relaxation of supporting structures and degeneration of articular cartilage^28^.

### *Sex Difference*

The “non‐uniform settlement” suggested that medial tibial plateau collapse was caused by long‐term compressive stresses on medial tibial plateau. *In vivo*, age represented the length of weightbearing on the bone. In our study, FBA and JLCA were significantly different between males and females (Table 2), that informed more obvious morphological changes that happened in females. According to statistics, females are at higher risk of osteoarthritis, osteoporosis, and autoimmune diseases than males^18^, ^19^, ^20^, ^21^, ^22^. Oestrogen deficiency and osteoporosis around the time of menopause might mainly contribute to this for intensification of the deformation accelerates the progression of KOA^34^. Our result showed there were significant correlations between age and all values in females. That is, with increasing age, the mechanical axis of the femur and tibia getting more and more varus (mMDFA and MPTA decrease), while the collapse of medial tibial plateau, relaxation of supporting structures, and degeneration of articular cartilage all progressed faster and faster (JLCA increase and min‐JSW decrease). A greater angle of valgus cut of the distal femur for TKA might be needed in older females, as FBA and DFVRA were also increased with age. Deformation and abnormal stress interacted and aggravated with each other. But in males, the significant difference was found only in min‐JSW variable (*P* < 0.01) (Table 4) and there was significant negative correlation between age and min‐JSW with *P* values less than 0.05 deemed significant (−0.134, *P* < 0.05) (Table 5). That is, the degeneration of articular cartilage may be the primary morphological change of male's lower extremities during medial compartment KOA, as no obvious dynamic deformations of bone were found. We consider the severity of degeneration of articular cartilage probably largely associated with the severity of congenital knee varus and age. In females, dynamic deformation accelerated the progression of articular cartilage's degeneration, that led to a more severe KOA. According to statistics, approximately 60% of TKA patients were females^35^, ^36^. Our practice shows 77.18% of the included knees were knees of females, the result confirm the susceptibility of females.

## Limitation of the Study

This study, which focuses on the morphology of KOA, is part of our series of studies in this area. The limitation of our research is: the effect of weight was not included in this study for this experiment is a retrospective study, and the X‐ray films which we collected did not contain body weight. Large‐scale prospective research and further biomechanical and clinical studies should be carried out to improve our theory.

### *Conclusions*

As medial compartment KOA progresses, dynamic deformation of bones of lower limbs and degeneration of articular cartilage could be found in females, while no obvious dynamic deformations of bone were found in males. Dynamic bone deformation may be an important cause of the high incidence and disability rate of KOA in female patients. Dynamic deformation of lower extremities was the important feature and the major causative factor of female KOA. We provided the theoretical basis for TKA and knee‐salvage treatment, that more attention should be paid to females in the preoperative protocol for orthomorphia, especially elderly females.

### *Authorship Declaration*

We declare that all authors listed meet the authorship criteria according to the latest guidelines of the International Committee of Medical Journal Editors, and that all authors are in agreement with the manuscript.
